# Pharmacokinetics and safety of bilastine in children aged 6 to 11 years with allergic rhinoconjunctivitis or chronic urticaria

**DOI:** 10.1007/s00431-019-03559-6

**Published:** 2020-01-09

**Authors:** Mónica Rodríguez, Valvanera Vozmediano, Aintzane García-Bea, Zoltán Novák, Anahí Yáñez, Cristina Campo, Luis Labeaga

**Affiliations:** 1 Drug Modeling & Consulting, Dynakin S. L., Bilbao, Spain; 2 Medical Department, FAES FARMA, S. A., 48940 Leioa, Bizkaia Spain; 3 Aranyklinika Egészségügyi és Innovációs Kft, Szeged, Hungary; 4 INAER-Investigaciones en Alergia y Enfermedades Respiratorias, Buenos Aires, Argentina

**Keywords:** Bilastine, Pharmacokinetics, Safety, Children, Allergic rhinoconjunctivitis, Urticaria

## Abstract

Bilastine, a second-generation antihistamine, is approved in Europe for the treatment of allergic rhinoconjunctivitis and urticaria in adults and children aged ≥ 6 years. Pharmacokinetic data for children aged 6–11 years were extracted post hoc from a study in which children (2–11 years) with allergic rhinoconjunctivitis or urticaria received oral bilastine (10 mg/day). Maximum plasma concentration (*C*_max_) and area under the plasma concentration curve (AUC) data were compared with adult pharmacokinetic data from seven clinical studies (bilastine 20 mg/day). Safety data for children aged 6–11 years were extracted post hoc from a phase III randomized controlled trial of children (2–11 years) with allergic rhinoconjunctivitis or chronic urticaria receiving once-daily bilastine 10 mg or placebo for 12 weeks. Exposure and *C*_max_ values were similar for children (6–11 years) and adults: median pediatric/adult ratios for AUC_0–24_ and *C*_max_ were 0.93 and 0.91, respectively. There was no significant difference in the incidence of treatment-emergent adverse in children (6–11 years) receiving bilastine 10 mg or placebo.

*Conclusion*: Pharmacokinetic and safety analyses in children aged 6–11 years support the suitability of the pediatric dose of bilastine 10 mg and confirm that the safety profiles of bilastine and placebo are similar.

**Table:** 

**What is Known:** • *Bilastine, a second-generation antihistamine, is approved in Europe for the treatment of allergic rhinoconjunctivitis and urticaria in adults (20 mg/day) and children aged ≥ 6 years (10 mg/day).* • *An ontogenic model based on adult data and pharmacokinetic/pharmacodynamic simulations supported the selection of a bilastine dose of 10 mg/day in children aged 2–11 years. Bilastine 10 mg/day was shown to have a safety profile similar to that of placebo in a large phase III randomized clinical trial in children aged 2–11 years.*	
**What is New:** • *As bilastine is approved in Europe for children aged ≥6 years, the current study reports the results of two post hoc analyses of pharmacokinetic and safety data in children aged 6–11 years.* • *Analysis of pharmacokinetic and safety data in children aged 6–11 years supports the suitability of the pediatric dose of bilastine 10 mg and confirms that its safety profile is similar to that of placebo.*

**What is Known:**

• *Bilastine, a second-generation antihistamine, is approved in Europe for the treatment of allergic rhinoconjunctivitis and urticaria in adults (20 mg/day) and children aged ≥ 6 years (10 mg/day).*

• *An ontogenic model based on adult data and pharmacokinetic/pharmacodynamic simulations supported the selection of a bilastine dose of 10 mg/day in children aged 2–11 years. Bilastine 10 mg/day was shown to have a safety profile similar to that of placebo in a large phase III randomized clinical trial in children aged 2–11 years.*

**What is New:**

• *As bilastine is approved in Europe for children aged ≥6 years, the current study reports the results of two post hoc analyses of pharmacokinetic and safety data in children aged 6–11 years.*

• *Analysis of pharmacokinetic and safety data in children aged 6–11 years supports the suitability of the pediatric dose of bilastine 10 mg and confirms that its safety profile is similar to that of placebo.*

## Introduction

Bilastine is a second-generation non-sedating and non-brain penetrating antihistamine which is approved in Europe for the treatment of allergic rhinoconjunctivitis and urticaria in adults and children aged ≥ 6 years with a body weight ≥ 20 kg [1–6]. Recently, bilastine has also been approved in Mexico for children aged ≥ 2 years. The efficacy of bilastine is similar to that of other second-generation oral H_1_-antihistamines [7–9].

Pharmacokinetic (PK)/pharmacodynamic (PD) modeling in healthy adult subjects, complemented with non-compartmental analysis, demonstrated linear kinetics of orally administered bilastine over a dose range of 2.5 to 220 mg [10]. In children aged 2 to 11 years, an ontogenic model based on adult data and PK/PD simulations supported the selection of a bilastine dose of 10 mg/day [11], which was confirmed in a clinical PK study of children aged 4 to 11 years with allergic rhinoconjunctivitis or urticaria [11, 12].

As bilastine is approved in Europe for children aged 6 years or over, the current study reports the results of two post hoc analyses of PK and safety data. PK data generated by Vozmediano and colleagues [12] were analyzed, focusing on children aged 6–11 years. Secondly, a post hoc analysis of safety data in the same age group was conducted. These data were obtained from a phase III, placebo-controlled, randomized, controlled trial of bilastine 10 mg/day for the treatment of children with allergic rhinoconjunctivitis or chronic urticaria [5].

## Methods

### Pharmacokinetic data

PK data were available from a multicenter, international, adaptive, open-label, repeated-administration study of oral bilastine 10 mg/day in children aged 4 to 11 years with allergic rhinoconjunctivitis or urticaria (BILA-3009/PED study; ClinicalTrials.gov Identifier: NCT01081574). The study has been described in detail elsewhere [12].

Previous PK population modeling of children used subjects aged 4–11 years [11, 12]. This post hoc analysis focuses on children aged 6–11 years (*n* = 24), in line with the approved pediatric indication in Europe.

#### Non-compartmental analysis

The maximum plasma concentration (*C*_max_) and area under the plasma concentration curve (AUC_0–24_) were calculated using S-PLUS® (Version 8.2, TIBCO Software, Palo Alto, CA, USA).

#### Comparison of NCA metrics in children with adults

Systemic exposure following oral bilastine pediatric dose (10 mg/day) was compared with adults (oral bilastine 20 mg/day) using numerical and graphical comparisons, including the use of a forest plot to visually explore inter-study heterogeneity. For pediatric data, bilastine plasma concentration (Cp) was plotted against time after dose (TAD) to account for sampling schedule differences.

Two alternative approaches were considered to establish reference AUC_0–24_ and *C*_max_ ranges in adults: the population-predicted 95% confidence interval (CI), based on the population PK model developed with adult data [10], and the more conservative 95% CI, based on NCA values obtained from seven Phase I studies (studies 459-02, 459-04 to 459-07, 459-10, and 459-11) in adults following bilastine 20 mg dosing.

### Safety data

Safety data were extracted from a phase III, double-blind, randomized, placebo-controlled, parallel-group study of children aged 2–11 years with allergic rhinoconjunctivitis or chronic urticaria treated once-daily with bilastine 10 mg (*n* = 260) or placebo (*n* = 249) for 12 weeks [5]. Full study details and results have been published previously [5].

Bilastine (10 mg) or placebo was administered orally once daily in the morning under fasting conditions for 12 weeks. Occasional use of rescue medication—short-term topical decongestants (eye/nose), corticosteroids or antihistamines for rhinoconjunctivitis, or short-term topical corticosteroids for urticaria was permitted.

The primary outcome was the proportion of children in each treatment group without any treatment-emergent adverse events (TEAEs; defined as any type of adverse events which occurred during the entire 4-month study duration (i.e., 3-month treatment period and 1-month follow-up period)), in accordance with PDCO guidance.

### Statistical analysis

Statistical significance was assessed for two-sided tests with an alpha of 0.05 as the cutoff for significance. Missing values were not considered for statistical calculations. Quantitative variables were described by the number of subjects, mean, standard deviation (SD), maximum, minimum, and quartile values. Qualitative variables were described by frequency and percentage. Secondary categorical variables were assessed using the chi-squared test or Fisher’s exact test if applicability conditions were not met.

## Results

### Pharmacokinetic data

**Fig. 1:**
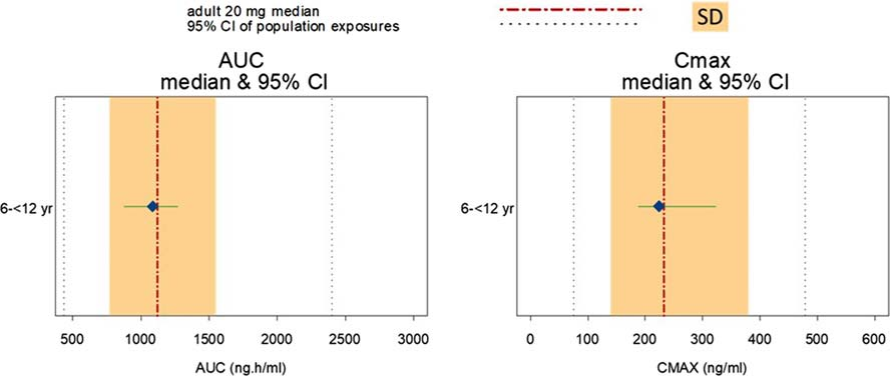
Forest plot for bilastine exposure (AUC) and *C*_max_ from observations in children (6–11 years old) in study BILA-3009/PED after the 10 mg dose and adults after 20 mg dose in several clinical studies (studies 459-02, 459-04 to 459-07, 459-10, 459-11). Blue dots represent the median values, and the green lines are the 95% confidence interval (95% CI) observed in children. The red line and the orange shaded area are the median value, and one standard deviation in the adult global population, respectively. In addition, the 95% CI of population exposures, as predicted by the population PK/PD model in adults after a 20 mg dose (435–2400 ng h/mL (AUC) and 75–475 ng/mL (*C*_max_)), are represented by the black dotted lines

A total of 88 samples from 24 children aged 6 to 11 years were collected, which enabled characterization of the absorption, distribution, and elimination phases of the PK profile in this cohort. Exposures and maximum plasma concentrations in the pediatric population (*n* = 12, corresponding to children with a rich PK profile in this sparse sampling design) after dosing with bilastine 10 mg/day were similar to those reported for adult PK data (*n* = 126) obtained from 7 clinical studies following daily dosing with bilastine 20 mg (Table 1). Median (SD) AUC_0–24_ in the pediatric and adult populations was 1045 (381) and 1121 (387) ng·h/mL, respectively, and median *C*_max_ was 212.0 (123) and 232.5 (120) ng/mL, respectively. Bilastine plasma concentrations observed in children completely overlapped those in adults, falling well within the adult variability, and followed the same PK temporal shape. Median pediatric/adult ratios for AUC_0–24_ and *C*_max_ were 0.93 and 0.91, respectively. Moreover, systemic exposure and *C*_max_ following oral bilastine pediatric dose (10 mg/day) was compared with adults (oral bilastine 20 mg/day) with the use of a forest plot (Fig. 1) showing that the pediatric PK metrics are completely inside the adult confidence intervals.

**Table 1:** Summary statistics for bilastine pharmacokinetic parameters AUC_0–24_ and *C*_max_ calculated in adults (bilastine 20 mg once daily) in seven clinical studies and in children aged 6–11 years (bilastine 10 mg once daily) from the PK study (BILA-3009/PED). Only children who had a complete PK profile were included (*n* = 12)

Parameter	Study	*N*	Mean	Median	SD	CV (%)	Range (min–max)	95% CI
AUC_0–24_ (ng·h/mL)	Adults (studies 459-02, 459-04 to 459-07, 459-10, 459-11)	126	1160	1121	387	33.4	491–2528	1092–1227
	Children aged 6–11 years (study: BILA-3009/PED)	12	1014	1045	381	37.6	363*–1653	798–1230
*C*_max_ (ng/mL)	Adults (studies 459-02, 459-04 to 459-07, 459-10, 459-11)	126	259.8	232.5	120	46.2	83–924	239–281
	Children aged 6–11 years (study: BILA-3009/PED)	12	239.2	212.0	123	51.5	61–447	170–309

*Minimum AUC value, identified in a single child and classed as a statistical outlier

*95% CI*, 95% confidence interval; *CV*, coefficient of variation; *SD*, standard deviation

### Safety data

The safety population comprised 393 subjects who received study medication and was randomized to receive bilastine 10 mg (*n* = 202) or placebo (*n* = 191). The mean (SD) age of the bilastine and placebo groups was 8.5 (1.6) and 8.5 (1.8) years, respectively. In the bilastine group, 105 (52%) subjects were aged 6–8 years, and 97 (48%) aged 9–11 years. Respective numbers in the placebo group were 95 (49.7%) and 96 (50.3%). Subjects in the bilastine and placebo groups were mainly male (62.9% and 61.8%, respectively) and Caucasian (93.1% and 92.7%). Mean (SD) body mass index (BMI) in the two groups was 18.0 (3.5) and 18.1 (3.5) kg/m^2^, respectively. Subjects were diagnosed with allergic rhinoconjunctivitis (98.0% and 93.7%) or chronic urticaria (2.0% and 6.3%), with a mean (SD) time since diagnosis of 4.1 (2.5) and 4.0 (2.7) years, respectively.

There was no significant difference in the incidence of TEAEs between the two groups: 137 events were reported in 67.8% of children receiving bilastine compared with 129 events in 67.5% receiving placebo (*p* = 0.952). Rates of children with related-TEAEs in the bilastine and placebo groups were 5.4% and 7.9%, respectively (*p* = 0.337); rates of children with serious TEAEs were 1.0% and 3.1%, respectively (*p* = 0.165), although none were considered to be treatment related; and rates of children with TEAEs leading to discontinuation were 1.0% and 0.5%, respectively (*p* = 1.0).

The most frequent TEAEs (≥ 5% frequency) in the bilastine and placebo groups were headache (13.4% vs. 12.6%), allergic conjunctivitis (9.9% vs. 9.4%), cough (8.9% vs. 7.9%), nasopharyngitis (7.9% vs. 4.7%), pharyngitis (7.4% vs. 6.8%), allergic rhinitis (6.4% vs. 10.0%), pyrexia (5.0% vs. 10.0%), and viral infection (4.5% vs. 5.2%), respectively.

## Discussion

Results from the post hoc PK study of bilastine in children aged 6–11 years with allergic rhinoconjunctivitis or urticaria align with those from a larger pediatric population (aged 2–11 years) which indicated a lack of age dependence for bilastine PK [11, 12]. The current PK analysis was limited to children aged 6–11 years, which is in line with the approved pediatric indication for bilastine in Europe: ≥ 6 years of age with a body weight of ≥ 20 kg. Pediatric data in the full dataset (aged 4–11 years) were best described by a two-compartment disposition model [11, 12], which is the same used to describe bilastine PK in adults [10]. Both AUC_0–24_ and *C*_max_ metrics between children (aged 6–11 years) and adult groups were very similar, with a complete overlap of pediatric bilastine plasma concentrations with those of adults, and pediatric/adult ratios for AUC_0–24_ and *C*_max_ were close to unity (0.93 and 0.91, respectively). These results support the suitability of the pediatric dose of bilastine 10 mg.

Safety data were derived from analysis of a subset of data (in children aged 6–11 years) from the phase III, placebo-controlled randomized trial of bilastine in children aged 2 to 11 years with allergic rhinoconjunctivitis or chronic urticaria [5]. In this post hoc analysis, there was no significant difference in the incidence of TEAEs in children receiving bilastine (10 mg) or placebo, with headache being the most commonly reported adverse effect in both groups (13.4% vs.12.6%, respectively). These results confirm that, in children, bilastine 10 mg has a safety and tolerability profile similar to that of placebo [5]. In adults, bilastine at the recommended dose of 20 mg has an excellent safety profile, with good tolerability and no sedative effects or cardiotoxicity [2, 7–9].

In conclusion, analysis of PK and safety data in children aged 6–11 years supports the suitability of the pediatric dose of bilastine 10 mg and confirms that its safety profile is similar to that of placebo.

## Abbreviations

AUC: Area under the plasma concentration curve
BMI: Body mass index
C_max_: Maximum plasma concentration
CI: Confidence interval
CV: Coefficient of variation
ECG: Electrocardiogram
NCA: Non-compartmental analysis
PD: Pharmacodynamic
PDCO: Pediatric Committee of the European Medicines Agency
PK: Pharmacokinetic
PSQ: Pediatric Sleep Questionnaire
PT: Preferred term
SD: Standard deviation
SOC: System Organ Class
TAD: Time after dose
TEAE: Treatment-emergent adverse event
